# How a single receptor-like kinase exerts diverse roles: lessons from FERONIA

**DOI:** 10.1186/s43897-022-00046-9

**Published:** 2022-11-18

**Authors:** Gaopeng Wang, Zhifang Zhao, Xinhang Zheng, Wenfeng Shan, Jiangbo Fan

**Affiliations:** 1 Shanghai Collaborative Innovation Center of Agri-Seeds, School of Agriculture and Biology, Shanghai Jiao Tong University, 800 Dongchuan Rd, Shanghai, 200240 China; 2 Joint Center for Single Cell Biology, School of Agriculture and Biology, Shanghai Jiao Tong University, 800 Dongchuan Rd, Shanghai, 200240 China

**Keywords:** FERONIA, Signaling pathway, Regulation mechanism, Immunity, Cell growth

## Abstract

FERONIA (FER) is a member of the *Catharanthus roseus* receptor-like kinase 1-like (CrRLK1L) protein subfamily, which participates in reproduction, abiotic stress, biotic stress, cell growth, hormone response, and other molecular mechanisms of plants. However, the mechanism by which a single RLK is capable of mediating multiple signals and activating multiple cellular responses remains unclear. Here, we summarize research progress revealing the spatial–temporal expression of FER, along with its co-receptors and ligands determined the function of FER signaling pathway in multiple organs. The specificity of the FER signaling pathway is proposed to operate under a four-layered mechanism: (1) Spatial–temporal expression of FER, co-receptors, and ligands specify diverse functions, (2) Specific ligands or ligand combinations trigger variable FER signaling pathways, (3) Diverse co-receptors confer diverse FER perception and response modes, and (4) Unique downstream components that modify FER signaling and responses. Moreover, the regulation mechanism of the signaling pathway- appears to depend on the interaction among the ligands, RLK receptors, co-receptors, and downstream components, which may be a general mechanism of RLKs to maintain signal specificity. This review will provide a insight into understanding the specificity determination of RLKs signaling in both model and horticultural crops.

**Supplementary Information:**

The online version contains supplementary material available at 10.1186/s43897-022-00046-9.

## Introduction

Receptor-like kinases (RLKs) and receptor-like proteins (RLPs) constitute the largest gene family in *Arabidopsis thaliana* and rice (*Oryza sativa*), with more than 600 and 1100 family members, respectively (He et al., 2018; Shiu and Bleecker 2003; Shiu et al., 2004). Compared with plants, animals have a much smaller number of RLKs and RLPs (Shiu and Bleecker 2001). An RLK is a transmembrane protein with an extracellular domain (ECD) and an intracellular kinase domain, which are separated by a single-pass transmembrane motif (Shiu and Bleecker 2003; Shiu et al., 2004). Owing to the high diversity in the structure of their ECDs, RLKs and RLPs are the most versatile plant gene families, enabling the recognition of a wide range of ligands (Lehti-Shiu et al., 2009). RLKs are categorized into multiple subfamilies based on their ECDs, including leucine-rich repeats, S-domain, legume lectin, wall-associated kinases, lysin motif domain, and *Catharanthus roseus* receptor-like kinase (CrRLK1L). As integral proteins localized on the plasma membrane, RLKs generally function as sensors to perceive external stimuli or as receptors to receive exogenous or endogenous signals (mostly chemicals or peptides). Upon the environmental or developmental stimulis are sensed by the ECDs of RLKs, the intracellular kinase domains will be activated to catalyze protein phosphorylation, which modulates protein activity, stability, and interactions (Cohen 2000). RLKs generally function as receptors or sensors with RLP partners, which depend on the kinase activity of RLKs to transduce a signal to downstream components.

Compared with RLKs, RLPs lack the kinase domain and possess only an ECD and transmembrane motif (Dievart et al., 2020). Different RLKs and/or RLPs possess varying degrees of perception specificity. Most RLKs and RLPs perceive and respond to very specific signals stringently. For example, BRASSINOSTEROID INSENSITIVE 1 (BRI1) perceives the endogenous steroid phytohormone brassinosteroid (BR), and FLAGELLIN-SENSING 2 (FLS2) perceives bacteria flagellin to trigger plant immune responses. In contrast, some RLKs and RLPs perceive multiple or even a wide array of ligands. For example, BRASSINOSTEROID INSENSITIVE1-ASSOCIATED KINASE1 (BAK1) participates in the perception of multiple signals, including BR, bacteria flagellin and EF-Tu, and several developmental peptides (Yang et al., 2011).

RLKs were also shown to facilitate the communication of cells with their environment for adaptation (Shiu and Bleecker 2003). RLKs participate in a broad array of biological processes in plants, including growth, development, morphogenesis, metabolism, reproduction, and responses to stresses (Yu et al., 2013). FERONIA (FER) is an important RLK in plants that perceives numerous types of endogenous signals [mostly rapid alkalinization factors (RALFs)] to participate in a series of physiological processes (Zhu et al., 2021). However, it remains unclear how a single RLK can achieve such versatile roles. From an informatic perspective, this question can be reframed as to how a single RLK is capable of sorting through numerous incoming signals precisely to respond accordingly. Determining the answers to these questions will provide deep insights into the specificity of signal discrimination. As the multiple roles of BAK1 in plant immunity have been discussed elsewhere (Yasuda et al., 2017), we here focus on the FER signaling pathways to address these questions, as these pathways have been intensely investigated with major insight gleaned in recent years. This review can help to further reveal the convergent and divergent signal transduction mechanisms in plants to improve understanding of the complexity and robustness of cell–cell and cell–environment communication.

## FER plays versatile roles in plants

*Arabidopsis* FER is the best-characterized member of the RLK subfamily CrRLK1L, which was first discovered in the synergid cells of the female gametophyte and was determined to be required for fertilization, the *fer* mutants show impaired fertilization, the pollen tube fails to arrest and thus continues to grow inside the femal gametophyte (Escobar-Restrepo et al., 2007). Subsequently, FER has been found to participate in many other essential biological processes, including reproduction modulation, responses to biotic and abiotic stress, phytohormone responses, growth and development regulation, and metabolic regulation (as summarized in Additional Table 1). Moreover, FER is ubiquitously expressed in different tissues, organs, and cell types in *Arabidopsis*, which is consistent with its versatile roles.

### Role of FER in fertilization

FER governs multiple cell–cell communication events in plant reproduction, including pollen-stigma recognition, pollen tube reception, polytubey block, and fertilization compensation. Upon pollination, the cysteine-rich POLLEN COAT PROTEIN B-class peptides (PCP-Bs) compete with papilla autocrine RALF23/33 for binding to the ANJEA (ANJ)–FER complex, which leads to a decline of stigmatic reactive oxygen species (ROS) and facilitates pollen hydration (Liu et al., 2021). After germination, the pollen tubes undergo tip growth to deliver two non-motile sperm to the ovule where they fuse with an egg and a central cell to achieve double fertilization (Johnson et al., 2019). Following pollen tube arrival, pollen tube-derived RALF4 and RALF19 bind to the FER–LORELEI (LRE) complex, which then recruits and activates NORTIA, a calmodulin-gated Ca^2+^ channel, to initiate Ca^2+^ spiking and further induce pollen tube rupture and sperm release (Gao et al., 2022).

To prevent lethality due to genome imbalance and chromosome segregation defects caused by polyspermy, RALF6, 7, 16, 36, and 37 peptide ligands are produced by the pollen tubes and perceived by the FER–ANJ/HERCULES RECEPTOR KINASE 1 (HERK1)–LRE complex to establish the polytubey block, a biological strategy to prevent entrance of supernumerary pollen tubes into the ovules, thus avoiding repeated fertilization (Galindo-Trigo et al., 2020; Zhong et al., 2022). Moreover, the linkage between FER and pectin transduces signals from the cell wall, and the de-esterified pectin takes part in establishment of the polytubey block through the FER signaling pathway (Duan et al., 2020).

When fertilization fails, the emergence of secondary pollen tubes is necessary to ensure reproductive success. Pollen tube rupture results in the loss of RALF peptides and the release of the polytubey block to allow the secondary pollen tubes to exit the septum as a mechanism of fertilization compensation (Zhong et al., 2022). This RALF6/7/16/36/37-mediated polytubey block and fertilization compensation represent a robust mechanism that allows for precise double fertilization in *Arabidopsis*.

### Roles of FER in the regulation of biotic and abiotic stress

The function of FER in regulating the response of plants to abiotic stress is well-characterized. Under salt stress, the FER–LORELEI-LIKE GPI-ANCHORED PROTEIN 1 (LLG1) complex perceives the pectin signal and monitors cell wall integrity to mediate salt stress-related responses (Zou et al., 2018). Under low-nitrogen conditions, the RPM1-INDUCED PROTEIN KINASE (RIPK)–FER–RALF1 complex phosphorylates TARGET OF RAPAMYCIN (TOR) kinase to promote growth of the true leaves, and treatment with specific amino acids (e.g., Gln, Asp, and Gly) was shown to increase the content of mature RALF1 (Song et al., 2022). Under phosphate starvation, PHOSPHATE STARVATION RESPONSE 1 (PHR1) binds to the promoters of *RALF* genes with a PHR1-binding site sequence (P1BS) and activates *RALF* expression. Induced RALF peptides are perceived by FER to inhibit plant immunity and enhance rhizosphere bacterial growth, which helps plants increase phosphate uptake and thus promote growth (Tang et al., 2022a). FER regulates Ca^2+^ signaling under mechanical stimulation and *fer* mutants exhibit defective growth responses to mechanical perturbation (Shih et al., 2014). FER also plays an important role in the temperature response. The *fer-ts* (temperature sensitive) mutants phenocopy wild-type *Arabidopsis* plants at normal temperature (20 °C), but show a growth defect at elevated temperature (30 °C), accompanied by rapid and specific inhibition of root hair initiation and elongation (Kim et al., 2021). The role of FER in establishing a molecular link between metal ion stress, growth, and cell wall integrity was also revealed (Richter et al., 2017).

FER interacts with FLS2/EF-TU RECEPTOR (EFR) to facilitate formation of the FLS2/EFR–BAK1 complex that initiates immune signaling (Stegmann et al., 2017). SITE-1 PROTEASE (S1P)-cleaved RALFs (RALF23/33) interact with FER, and restrain the interaction between FER and FLS2/EFR to inhibit immunity (Stegmann et al., 2017). This mechanism is also utilized by pathogens and nematodes to inhibit plant immunity (Masachis et al., 2016; Zhang et al., 2020a). For example, *Fusarium oxysporum* secretes RALF-like (F-RALF) peptides to hijack the plant FER pathway, which induces the alkalinization of apoplasts and activates the orthologous mitogen-activated protein kinase (MAPK) FMK1 to promote fungal virulence (Masachis et al., 2016). Similarly, the nematode *Meloidogyne incognita* responsible for plant root knot secretes MiRALF1 and MiRALF3 to facilitate parasitism in a FER-dependent manner (Zhang et al., 2020a).

### Roles of FER in hormone signaling

Hormone regulation is one of the most important signaling pathways that FER participates in to modulate plant growth and development. RALF1–FER promotes YUCCA expression to initiate auxin biosynthesis, and induces the canonical TRANSPORT INHIBITOR RESPONSE1 (TIR1) and AUXIN-SIGNALING F-BOX (AFB) transcriptional pathways for sustained root growth inhibition within approximately 1 h from stimulation (Li et al., 2022a). FER is also involved in polar auxin transport, and *Arabidopsis fer* mutants show aberrant localization of PIN-FORMED2 (PIN2) (Dong et al., 2019). In addition, mutating the *PIN2* or *AUXIN RESISTANT1(AUX1)* gene was found to suppress polar auxin transport and caused asymmetric root growth in *fer* mutants (Li et al., 2020). The RALF1–FER pathway also initiates the GEF1/4/10–ROP11 pathway to activate ABA INSENSITIVE 2 (ABI2) phosphatase and inhibit the abscisic acid (ABA) response (Chen et al., 2016). Coronatine (COR) is a phytotoxin that mimics jasmonic acid (JA), and compromises host immunity by utilizing the transcription factor MYC2 to activate NAC transcription factors and further inhibit the accumulation of salicylic acid, leaving the host susceptible to disease (Xin and Sheng Yang 2013; Zheng et al., 2012). FER was reported to phosphorylate and destabilize MYC2 in regulating COR-mediated host disease susceptibility (Guo et al., 2018). The expression level of FER was shown to be positively regulated by ethylene, and ethylene-inducible hypocotyl growth could be inhibited by mutating FER. Interestingly, the effect of ethylene on hypocotyl growth could be antagonized by BR-mediated FER signaling (Deslauriers and Larsen 2010). BR was also reported to function antagonistically with RALFs (Bergonci et al., 2014a; Bergonci et al., 2014b; Srivastava et al., 2009). BR-specific phenotypes such as hypocotyl elongation and root growth could be compromised by RALF23 treatment (Srivastava et al., 2009). Conversely, brassinolide treatment inhibited the expression of RALF23 (Srivastava et al., 2009). Overexpressing AtRALF1 induced the expression of BR-downregulated genes and led to BR insensitivity; AtRALF1-induced gene expression could also be reduced by the treatment of exogenous brassinolide (Bergonci et al., 2014a). Moreover, FER negatively regulates S-adenosylmethionine (SAM) synthesis to downregulate ethylene biosynthesis through interacting with SAM1 and SAM2 (Mao et al., 2015).

### Roles of FER in other signaling pathways

Recent studies have revealed multiple additional biological processes modulated by FER. FER was reported to negatively regulate endoplasmic reticulum (ER) body formation and indolic glucosinolate biosynthesis through negative regulation of the transcription factor NAI1. Moreover, FER is required for TOR kinase activity, which negatively regulates autophagy (Wang et al., 2022b). RALF1/33/36 reversibly inhibits primary root growth through apoplast alkalinization within 1 min through the FER pathway (Gjetting et al., 2020; Li et al., 2022b). FER also interacts with the blue-light receptor PHOTOTROPIN 1 (PHOT1) and participates in PHOT1-mediated phototropic cell growth regulation (Li et al., 2022a). Cell wall pectin interacts with FER to activate the ROP6 guanosine triphosphatase (GTPase) signaling pathway that regulates formation of the puzzle-piece shape of pavement cells (PCs) in *Arabidopsis* (Tang et al., 2022b). FER also phosphorylates and destabilizes ABA INSENSITIVE 5 (ABI5), an important transcription factor in ABA signaling, to negatively mediate cotyledon greening (Wang et al., 2022a).

## Mechanisms regulating the FER signaling specificity

**Fig. 1:**
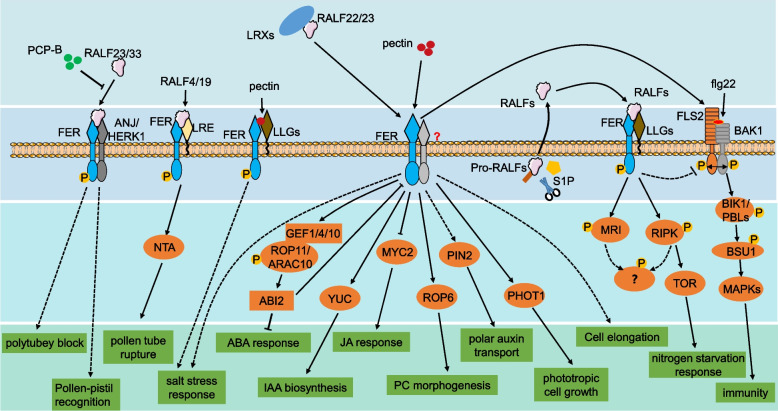
Model of FER signaling pathways. The FER signaling pathway could be modulated by a 4-layers mechanism. Firstly, the extracellular ligands (RALFs, pectin and PCP-Bs) and ligand combinations (LRXs) could trigger relatively specific FER signal activation. PCP-Bs compete with RALF23/33 to deactivate FER regulated polytubey block and pollen-pistil recognition. RALF4/19 and pectin activate FER mediated pollen tube rupture and salt stress response, respectively. RALF22/23 prefer to interact with cell wall-associated LRXs at acidic pH, and RALF22/23-LRXs complex could be disassociated under salt stress or alkaline conditions to release RALF22/23 and activate FER signaling pathway. Secondly, the diverse co-receptors(ANJ/HERK1, LRE and LLGs) provide diverse perception and responses mode for FER. FER forms a complex with ANJ/HERK1 to sense PCP-Bs and RALF23/33 signals. The FER-RALF4/19-LRE complex was formed upon recognizing RALF4/19 ligands. Moreover, FER form complexes with LLGs to sense pectin and RALFs. The third FER signaling modification layer was compromised of downstream components of FER. NTA, YUC, MYC2, ROP6, PIN2 and PHOT1 are downstream components for FER, which mediate FER regulated pollen tube rupture, IAA biosynthesis, JA response, PC morphogenesis, polar auxin transport and phototropic cell growth, respectively. The FER downstream GEF1/4/10-ROP11-ABI2 pathway was also revealed to regulate ABA response. MRI and RIPK are two RLCKs downstream of FER and RIPK directly interact with TOR to regulate FER related nitrogen starvation response. However, more RLCKs downstream of FER and more pathways downstream of the RLCKs remain to be discovered. Moreover, FER interacts with FLS2 to facilate the formation of FLS2-flg22-BAK1 complex and thus activate FLS2-BIK1-BSU1-MAPKs mediated immunity responses

As FER shows different functions in diverse signaling pathways (Additional Table 1), it is intriguing to question how a single RLK can mediate the multiple signals summarized above and appropriately regulate diverse plant physiological processes. To effectively and precisely regulate target signal pathways under certain conditions, the activation and deactivation of FER should be accurately modulated. Based on current findings, the specificity of FER functions can be attributed to four levels of regulation, as schematically depicted in Fig. 1: (1) spatial–temporal expression of FER co-receptors and/or ligands, (2) specific ligands or ligand combinations, (3) diverse receptor and co-receptor complexes on the plasma membrane, and (4) unique downstream signaling pathways. Each of these regulation mechanisms is discussed in turn below.

### Spatial–temporal expression of FER, co-receptors, and ligands specify diverse functions

In general, transcriptional regulation is the primary regulating step for protein kinases (Misra et al., 2002). In *Arabidopsis*, *FER* mRNA expression was found to be up-regulated by hormones that typically play a positive role in cell growth (i.e., auxin) (Guo et al., 2009; Yu et al., 2012), which in turn suppressed the response of hormones that negatively regulate cell growth (i.e., ABA) (Deslauriers and Larsen 2010; Yu et al., 2012). Indole-3-acetic acid (IAA) induced upregulated expression of *FER* mRNA and directly facilitated FER-regulated Rho GTPase signaling in root hair development (Duan et al., 2010). Deslauriers and Larsen (2010) reported that ethylene treatment induced the expression of FER and that the FER-mediated BR response antagonized the effect of ethylene on the hypocotyl growth of etiolated seedlings. Yu et al., (2012) reported that ABA downregulates the expression of *FER* mRNA, and FER negatively regulates ABA-induced root growth suppression or stomatal closure by activating the guanine nucleotide exchange factors (GEFs)–Rho complex of the PLANT 11 (ROP11) pathway to ultimately enhance the activity of the phosphatase ABI2, a negative regulator of the ABA signaling pathway.

The spatial–temporal expression and localization of FER, co-receptors, and their ligands (RALFs) are vital for FER functions with different specificities. The mRNA of *FER* was detected in floral apices, young ovule primordia, and young anthers with immature pollen, but was not detected in mature pollen or older anthers harboring mature pollen (Escobar-Restrepo et al., 2007). Pollen coat-derived PCP-B peptides compete with papilla cell autocrine RALF23/33 for interaction with the ANJ–FER complex, repressing ROS production and initiating stigmatic responses that lead to self-pollen germination. FER was also reported to be localized in the filiform apparatus of synergid cells, where it binds the putative ligand on the approaching male gametophyte (pollen tube). The interaction between the putative male ligand and the ECD of FER triggers a signal transduction cascade inside the synegid cell; a subsequent signal then feeds back from the synergid cell to the pollen tube, causing growth arrest and the release of the sperm cells (Escobar-Restrepo et al., 2007). When FER is mutated, the pollen tube fails to arrest and thus continues to grow inside the female gametophyte (Escobar-Restrepo et al., 2007). Duan et al., (2014) further revealed that FER positively regulates the production of ROS at the entrance to the female gametophyte to induce pollen tube rupture and sperm release, which is consistent with the expression pattern of FER in the reproductive organs.

Thus, the expression and localization of FER in flowers appear to determine its function in fertilization in *Arabidopsis*. In addition to its expression in the ovule, FER is broadly expressed in the leaves, buds, flowers, siliques, and roots, and the function of FER in plant morphology establishment is largely dependent on its localization and expression in the roots and shoots (Duan et al., 2010; Escobar-Restrepo et al., 2007). Duan et al., (2010) revealed that FER is localized to the plasma membrane of the root hairs and positively regulates IAA-induced RAC/ROP-regulated ROS production while mediating root hair growth. Consistently, IAA-induced root hair development is significantly repressed in *fer-4* mutants. Similarly, the mRNA level of *FER* in *Arabidopsis* shoots is largely related to seedling morphogenesis. Ethylene negatively regulates cell growth, and the *fer-2* mutants showed an ethylene-sensitive phenotype with significantly smaller rosettes and a severe hypocotyl shortening phenotype (Deslauriers and Larsen 2010). Conversely, the root growth in *fer-2* mutants showed an ethylene-insensitive phenotype, which implies that FER regulates the ethylene pathway in an organ-specific manner (Deslauriers and Larsen 2010). In contrast to its function in promoting root cell growth, FER was found to negatively regulate the elongation of integument cells, thereby restraining the seed size (Yu et al., 2014).

The subcellular location of FER also plays an important role in FER-regulated signaling pathways. LLG1 is a FER co-receptor that is required for the transport of FER from the ER to the plasma membrane. Mutation of *llg1* resulted in cytoplasmic retention of FER, leading to indistinguishable growth, developmental, and signaling phenotypes (Li et al., 2015). Mature RALF22/23 peptides or salt stress could induce the internalization of FER and negatively regulated the function of FER in salt tolerance (Zhao et al., 2018). Moreover, a kinase-dead mutation of FER (FER^K565R^) fully complemented the deficiency of ovule fertilization in *fer-4* mutants, but simultaneously reduced the ability to complement the root responses to RALF1 and RALF1-induced cytoplasmic calcium mobilization (Haruta et al., 2018). These findings support that the cell type-specific regulatory mechanisms of FER result from its different interacting partners and/or downstream signaling events.

Taken together, the diverse functions of FER appear to depend, at least in part, on the spatial–temporal expression of FER, co-receptors, and ligands in distinct cells or organs.

### Specific ligands or ligand combinations trigger variable FER signaling pathways

FER signaling pathways are triggered by interaction with its ligands or ligand combinations, which could also play important roles in FER-involved pathways. FER belongs to the CrRLK1L family of RLKs and is characterized by a malectin-like ECD, which enables the perception of plant cysteine-rich peptides of the RALF family (DeFalco 2022; Zhu et al., 2021). In addition to perceiving peptides, FER also acts as a receptor for flower-specific PCP-B (Liu et al., 2021), cell wall pectin (Tang et al., 2022b), and RALFs-mediated leucine-rich repeat extensin (LRX) signals (Herger et al., 2019).

RALFs constitute an evolutionarily conserved peptide family, with at least 37 RALF members identified in *Arabidopsis* to date*.* According to Campbell and Turner (2017), RALFs could be divided into four major clades: clades I, II, and III contain an RRXL cleavage site, a SITE-1 PROTEASE (S1P) site, and the YISY motif required for receptor binding, whereas clade IV RALFs are highly divergent and lack these typical RALF sites. In general, RALFs initiate FER-dependent downstream phosphorylation signal cascades (Haruta et al., 2014; Zhang et al., 2020b). Xiao et al., (2019) revealed that a conserved N-terminal region of RALF23 is sufficient to activate FER phosphorylation, and the interaction was reinforced by the C-terminal region of RALF23. Although the oldest *RALF* genes were found in the non-flowering plant *Physcomitrella patens*, *RALF* genes have also been identified outside of the Plant kingdom. The evolutionary history of RALF peptides appears to be more complex than that of the CrRLK1L family (Campbell and Turner 2017). Except for pollen-specific RALF4, other RALF peptides tested to date have the ability to promote extracellular alkalinization (Morato do Canto et al., 2014). Among the *Arabidopsis* RALFs, 11 RALFs (RALF1/9/14/18/22/23/33/27/31/34) display a S1P cleavage site, which cleaves endogenous RALF pro-peptides (Pro-RALFs) to produce mature RALFs that inhibit plant immunity in a FER-dependent manner. FER facilitates pathogen-associated molecular pattern (PAMP)-induced complex formation of the immune receptor kinases EFR and FLS2 with their co-receptor BAK1 to initiate immune signaling without RALFs perception (Stegmann et al., 2017; Tang et al., 2022a). By contrast, RALFs lacking a predicted S1P cleavage site only participate in growth regulation but do not play a role in immune manipulation (Haruta et al., 2014; Stegmann et al., 2017). Treatment with RALFs possessing an S1P site (e.g., RALF23 and RALF33) not only triggered seedling growth inhibition but also suppressed an ELF18-induced ROS burst, whereas treatment with RALFs without an S1P site (e.g., RALF1 and RALF32) could only trigger seedling growth inhibition (Stegmann et al., 2017). As all of these RALFs commonly utilize FER as a receptor, and only the S1P-cleaved RALFs could inhibit plant immunity, we propose that the proteolytic cleavage mechanism could serve as a specific signal to distinguish the immunity signal and growth-related regulation.

PCP-Bs are small cysteine-rich peptides expressed in the maturing pollen coat and participate in pollen–pistil recognition. The *pcp-b* mutants show impaired pollen hydration, reduced pollen adhesion, and delayed pollen tube growth (Wang et al., 2017). A recent study revealed that PCP-B peptides could function as ligands for FER receptor to facilitate pollination, and PCP-Bs also compete with RALF23/33 to interact with the ANJ–FER receptor complex, thereby inhibiting ROS production to initiate stigmatic responses upon pollination (Liu et al., 2021).

Pectins are important structural polysaccharides in cell walls that can be demethylesterified by pectin methyl esterases (Sénéchal et al., 2015). FER recognizes demethylesterified pectin through the malectin A domain, and directly interacts with ROP–GEF14, followed by activating the intracellular ROP6 GTPase signaling pathway to regulate the formation of the jigsaw cell shape in the leaf epidermis (Tang et al., 2022b). The interaction between pectin and FER links the cell wall signal to appropriate intracellular responses.

LRXs are chimeric proteins that are insoluble in the cell wall, which can form protein–protein interaction platforms (Herger et al., 2019). Although LRXs are not direct ligands for FER, they were found to interact with FER and convey extracellular signals to the cell by forming complexes with RALF ligands (Dünser et al., 2019; Herger et al., 2020). LRXs commonly possess a C-terminal extensin domain and a leucine-rich repeat N-terminal domain with high affinity for RALFs binding (Mecchia et al., 2017). The interaction between RALF1 and LRX3/4/5 was reported in both the shoots and roots to regulate cell wall signaling and plant growth; LRX3/4/5 links plasma membrane-localized FER with the cell wall, allowing this module to jointly sense RALF signals. This interaction coordinates the onset of cell wall acidification and loosening with an increase in vacuolar size (Dünser et al., 2019). A direct interaction between RALF4 and LRXs was also revealed, in which the LRXs are necessary for RALF4 signaling to maintain cell wall integrity during pollen tube growth (Mecchia et al., 2017). LRX3–5 directly interact with RALF22 and RALF23, and further associate with plasma membrane-localized FER to participate in the salt stress response. Salt stress could promote the release of mature RALF22 through S1P cleavage. Both salt stress and RALF22/23 treatment facilitated disassociation of the LRXs–RALF complex and the internalization of FER (Zhao et al., 2018). Moreover, overexpression of RALF22/23 resulted in retarded growth and salt hypersensitivity, as found in *lrx3/lrx4/lrx5* triple mutants and *fer* mutants (Zhao et al., 2018). Interestingly, the cell wall pH condition could alter the affinity between RALFs and their binding proteins: RALFs showed high binding affinity with LRXs at acidic pH, but preferentially bound to LORELEI-LIKE GPI-ANCHORED PROTEINS (LLGs) under neutral/alkaline conditions. This indicates that plant growth and immunity regulation could be related to cell wall pH modulation (Moussu et al., 2020). Moreover, the loop region in RALFs (e.g., residues 70 to 101 in RALF4) show high binding affinity with LRXs, and the N-terminal alpha-helix (e.g., residues 64 to 69 in RALF4) of RALFs is a major determinant for RALFs–LLGs binding (Xiao et al., 2019).

The specific ligands or ligand combinations triggering FER signaling pathways are present throughout the plant. In flower organs, different origins of FER ligands or ligand combinations define their specific roles in plant reproduction. RALF4/19 secreted by the pollen tubes are involved in pollen tube rupture and sperm release (Gao et al., 2022). Ovular pectin- and pollen tube-derived RALF6/7/16/36/37 are involved in the polytubey block (Duan et al., 2020; Galindo-Trigo et al., 2020; Zhong et al., 2022). Pollen coat-derived PCP-Bs and papilla-secreted RALF23/33 are responsible for pollen–pistil recognition (Liu et al., 2021). In the leaves, different RALF members exhibit varying functions. RALF1 promotes the growth of true leaves and confers tolerance to a nitrogen-starvation condition (Song et al., 2022). RALFs with an S1P site participate in FLS2/EFR-mediated immune responses (Stegmann et al., 2017). RALF1/23 are involved in the responses to JA and BR stimuli (Bergonci et al., 2014a; Bergonci et al., 2014b; Guo et al., 2018; Srivastava et al., 2009). Pectin derived from the cotyledon epidermal PCs regulates the morphogenesis of PCs via FER signaling (Tang et al., 2022b). In the roots, multiple ligands are involved in stress responses and growth regulation. The pectin-, LRX3/4/5-, and RALF22/23-meditated FER signaling pathways are involved in salt stress (Zhao et al., 2018; Zou et al., 2018). RALFs that containing a P1BS motif are could response to phosphate starvation signals (Tang et al., 2022a). The RALF1/22/33/36-triggered FER signaling pathway inhibits root elongation (Gjetting et al., 2020; Li et al., 2022a). RALF1 inhibits the ABA response (Chen et al., 2016), and RALF1/22 promote auxin biosynthesis and the IAA response (Li et al., 2022b).

The fact that multiple ligands trigger diverse FER signaling pathways indicates that the first layer in FER signaling pathway regulation depends on the diversity of ligands and ligand–receptor two-way selection.

### Diverse co-receptors confer diverse FER perception and response modes

The two-way selection pattern of ligands–receptor–co-receptor complexes provide diverse responses modes for FER signaling pathways. Upon the perception of ligands, RLK receptors typically form complexes with co-receptors to activate corresponding signaling pathways (Couto and Zipfel 2016). For example, the co-receptor BAK1 associates with the receptor FLS2 to perceive the flagellin peptide flg22 and activate PAMP-triggered immunity (PTI) responses in *Arabidopsis* (Robatzek and Nürnberger 2007). FLS2–BAK1 heterodimerization occurs almost instantly following flg22 perception, suggesting that RLKs and their co-receptors might already be present at the plasma membrane in the form of pre-assembled inactive complexes (Couto and Zipfel 2016). As in FER-mediated signaling pathways, LRE, LLGs, and some CrRLK1s function as co-receptors for ligand-triggered FER activation (Feng et al., 2018; Galindo-Trigo et al., 2020; Gao et al., 2022; Zhong et al., 2022).

LRE is mainly expressed in the synergid cells of the ovule and is required for FER-mediated pollen tube burst (Liu et al., 2016). A recent study revealed that LRE is responsible for activating the FER signaling pathway that mediates pollen tube rupture and sperm release (Gao et al., 2022). LRE also forms a heterotetramer with the FER–ANJ–HERK1 complex to establish the polytubey block (Galindo-Trigo et al., 2020; Zhong et al., 2022). As LRE homologs, LLGs are the most common co-receptors for the RLK FER. LLGs directly interact with the ECD of FER to form the RALFs–LLG–FER complex and regulate FER signaling pathways (Li et al., 2015; Xiao et al., 2019). LLG1 directly interacts with FER not only on the cell surface but also in the ER; in *llg1* mutants, FER fails to localize at the plasma membrane and is retained in the ER, indicating that the subcellular localization of FER depends on formation of the FER–LLG1 complex (Li et al., 2015). Moreover, LLG1 is a component of the FER-regulated Rho GTPase signaling complex, and *llg1* mutants show similar growth and developmental phenotypes to those of *fer* mutants (Li et al., 2015). Further research revealed that RALFs prefer binding to cell wall-associated LRX proteins at acidic pH, but exhibit high binding affinity with membrane-localized LLGs under neutral or alkaline conditions (Moussu et al., 2020). Interestingly, all RALF peptides except for the pollen-specific RALF4 could promote extracellular alkalinization (Morato do Canto et al., 2014). Haruta et al., (2014) revealed that RALF1 peptides initiate FER kinase activation to inhibit H^+^-ATPase activity, and trigger the phosphorylation of plasma membrane-localized H + -adenosine triphosphatase 2 (H^+^-ATPase 2, AHA2) at Ser^899^ to restrain proton transport, leading to the occurrence of extracellular alkalinization and suppression of root elongation. Therefore, RALF-induced pH alteration is proposed to be a mechanism through which LLGs–RALFs participate in FER signaling pathways.

Apart from forming a complex with FER, LLGs also form different receptor–co-receptor complexes with other members of the CrRLK1L subfamily [i.e., ANXUR (ANX) and BUDDHA’S PAPER SEAL (BUPS)] to participate in RALF4 and RALF19 peptides-mediated ROS production, pollen tube growth, and cell wall integrity maintenance (Feng et al., 2019; Ge et al., 2017; Zhu et al., 2021). Similar to their function in FER subcellular localization, LLGs help ANX/BUPS localize on the cell membrane; both *llg2/3* and *anx1/2* mutants lacking the J region (the ANX/BUPS–LLG2/3-interacting region) exhibited cytoplasmic retention of ANX1/2 (Feng et al., 2019). The ANX/BUPS–LLGs complex may participate in perception of the RALF4 signal to control the maintenance of pollen tube integrity. RALF4 blocks wild-type pollen germination, but does not impact the pollen grains from *bups1*, *bups2*, *llg2*, or *llg3* mutants (Dünser et al., 2019). Further research revealed that BUPS1/2 could form a complex with GEF1/12 and ROP1/3/5/9 (Zhu et al., 2018), and the RALF4–ANX/BUPS–LLGs complex regulates pollen tube rupture/burst growth through the GEF–RAC/ROP–RBOH module (Feng et al., 2019). Ge et al., (2017) revealed that BUPS1/2, ANX1/2, and RALF4/19 directly interact, whereas RALF34 interacts with and competes for the RALF4/19 binding site in the ANX–BUPS receptor complex at the interface of the pollen tube, thereby deregulating the BUPS–ANX signal to promote pollen tube rupture and sperm release.

ANJ is a CrRLK1L family receptor kinase expressed in the filiform apparatus of the synergid cells of unfertilized ovules and stigma papilla cells (Galindo-Trigo et al., 2020). ANJ promotes pollen tube growth arrest redundantly with HERK1. The *herk1/anj* double mutant exhibits pollen tube overgrowth, leading to an unfertilized ovule (Galindo-Trigo et al., 2020). Upon perceiving the ligands RALF22/23, the activated ANJ–FER receptor complex on papilla cells induces ROS production through the ANJ–FER–ROP2–RESPIRATORY BURST OXIDASE HOMOLOGUE D (RBOHD) module before pollination, which could be suppressed by pollen coat PCP-B peptides (Liu et al., 2021).

As multiple CrRLK1L proteins (e.g., FER, ANX1/2, and BUPS1/2) may compete for LGG co-receptors, and multiple co-receptors (LRE, LLGs, and CrRLK1Ls) can form heterotrimers with the FER–ligand complex, we propose that different affinities between receptors and co-receptors could initiate various signaling processes, thus offering a potential regulation mechanism for the FER signaling pathway.

### Unique downstream components that modify FER signaling

As FER is not the direct executor of the multiple signaling pathways it participates in, downstream components are required to transduce FER signaling or execute specific FER-mediated cellular responses. RLCKs are direct downstream components of RLKs-regulated signaling pathways (Couto and Zipfel 2016). For example, the activation of FLS2 and BAK1 further phosphorylates RLCK VII–BOTRYTIS-INDUCED KINASE 1 (BIK1) to initiate the disassociation of BIK1 from the FLS2–BAK1–BIK1 complex and activate downstream signaling components (Zou et al., 2018). Similarly, FER is phosphorylated upon perceiving RALF ligands, and the phosphorylation state is further transduced to downstream RLCKs for signal activation (Du et al., 2016).

The plasma membrane-associated RIPK in the RLCK-VII family and MARIS (MRI) in the RLCK-VIII family are two recognized downstream RLCKs in the FER-regulated root growth signaling pathway (Boisson-Dernier et al., 2015; Du et al., 2016). RIPK interacts with FER to form a protein kinase complex, which phosphorylate each other in a mutually dependent manner at the plasma membrane (Du et al., 2016). The formation and phosphorylation of the FER–RIPK complex could be enhanced by RALF1 to positively regulate the RALF1 response in the roots (Du et al., 2016). However, *ripk* mutants did not show the notable pollen tube reception or seed size control phenotypes observed in *fer-4* mutants, indicating that there could be additional downstream RLCKs that fulfill distinct functions in the FER signal pathway. MRI is mainly expressed in the pollen tubes and root hairs, controlling cell wall integrity downstream of ANX1/2 and NADPH oxidases in the pollen tubes, and functions downstream of FER in the root hairs. The *mri* mutants display spontaneous pollen tube formation, similar to *anx1/anx2* mutants, and root-hair bursting, similar to *fer* mutants (Boisson-Dernier et al., 2015). Moreover, FER regulates the pollen tube burst, ABA response, IAA polar transport, IAA response, JA response, PC morphogenesis, and phototropic cell growth through the function of NORTIA (Gao et al., 2022), GEF1/4/10-ROP11 (Chen et al., 2016), PIN2 (Li et al., 2020), YUCCA (Li et al., 2022a), MYC2 (Guo et al., 2018), ROP6/GTPase (Tang et al., 2022a), and PHOT1 (Li et al., 2022a), respectively (Fig. 1). Although the functions of RLCKs in these processes have not been reported, we propose that RLCKs play vital roles in the described FER-mediated signaling pathways, which warrant further investigation.

Apart from the phosphorylation and dephosphorylation interactions between proteins, the link between protein kinases and distinct cellular responses could also be regulated by phosphocodes. In *Arabidopsis*, BRI1 regulates the activity of downstream GLYCOGEN SYNTHASE KINASE 3 (GSK3) and the flg22 receptor FLS2 regulates downstream MAPKs activation (Li et al., 2002; Robatzek and Nürnberger 2007). The activation of BRI1 and FLS2 involves a common co-receptor, BAK1. Upon recognizing the exogenous signals, the flg22 signal is further transduced to BIK1 and PBS1-LIKE, and the BR signal is transduced to BR signaling kinases and CONSTITUTIVE DIFFERENTIAL GROWTH 1 (Li et al., 2002; Robatzek and Nürnberger 2007). BIK1 phosphorylates BRI1-SUPPRESSOR 1 (BSU1) at Ser^251^ upon flg22 recognition to regulate downstream MAPK activation, whereas BR signaling kinases phosphorylate BSU1 at Ser^764^ upon BR recognition to inhibit GSK3. BSU1^S251A^ mutants showed reduced flagellin-induced MAPK activation and immunity, whereas the effector-triggered immunity and interaction between BSU1 and GSK3 were maintained (Park et al., 2022). Conversely, BSU1^S764A^ mutants induced the interaction between BSU1 and GSK3, and the MAPK activation and PTI response were maintained (Park et al., 2022). This implies a possible mechanism by which shared downstream components confer signaling specificities for diverse protein kinases with different phosphorylation sites.

## Conclusion and perspectives

To understand how a single RLK plays versatile roles in plants, we focused on the well-studied *Arabidopsis* CrRLK1L member FER to propose the mechanisms and strategies employed to achieve its diverse functions. First, regulation of the spatial–temporal expression and subcellular localization of FER, its co-receptors, and ligands in distinct cells or organs appear to determine the specific functions of FER in multiple organs. When FER is expressed and localized on the plasma membrane, multiple ligands (RALFs, pectin, PCP-Bs, and perhaps others that have yet to be discovered) could be perceived by FER to activate specific FER signaling pathways in a ligand–receptor-specific manner. Upon perceiving ligands, co-receptors are necessary for the activation of FER. Multiple co-receptors (LRE, LLGs, and CrRLK1Ls) could form heterotrimers with the FER–ligand complex for FER activation, and multiple CrRLK1L proteins (e.g., FER, ANX1/2, and BUPS1/2) compete for the binding site of co-receptors; thus, the affinities among ligands, receptors, and co-receptors determine the specificity of activated signaling pathways. After activation, FER transduces the signal through the activation of downstream RLCKs or other signaling components (Fig. 1); thus, proper activation of corresponding RLCKs or other factors is a vital regulation step for the FER signaling pathway. Although only two RLCKs acting downstream of FER have been discovered to date, we propose that there are likely more RLCKs downstream of FER. Therefore, the signaling specificity can be determined at multiple levels involving ligands, RLK receptors, RLP co-receptors, and downstream components, which combine to achieve diverse and specific functions.

Despite extensive research on the diverse functions of FER, the specificity of FER signaling pathways remains poorly understood. After activation, FER signaling is further transduced to downstream RLCKs, and different downstream RLCKs could provide divergent signaling and responses. RLCKs broadly function downstream of the RLK receptor and upstream of responsive components (Liang and Zhou 2018). As RIPK and MRI RLCKs are the only effectors acting downstream of FER studied to date, discovering more downstream RLCKs should be an area of focus in further research to better understand FER signaling pathways and functions, since RLCKs are highly conserved signaling components acting directly downstream of receptor complexes (Liang and Zhou 2018). Moreover, the opposite roles of FER monomer and the FER–RALF23–LLGs complex in immunity regulation requires more in-depth investigation. In particular, the mechanism by which the FER–RALF23–LLGs complex disassociates the FLS2–BAK1 complex and the role of FER signaling pathway-mediated regulation of the “trade-off” between growth and immunity remain to be elucidated. Apart from interacting with RLP co-receptors, FER may also interact with multiple membrane-localized RLKs to achieve its functions, as evident by the formation of complex receptor networks on the plasma membrane. Thus, it will be interesting to investigate how FER interacts with other RLKs, and identifying the specific RLKs involved in FER-mediated signaling will provide further insight into FER functional diversity and specificity.

### Supplementary Information

**Additional file 1: Additional Table 1.** Function of FER with corresponding ligands and coreceptors.

## Data Availability

Not applicable to this article as no datasets were generated or analyzed during the current study.

## Abbreviations

ABA: Abscisic acid
ABI2: ABA Insensitive 2
ABI5: ABA Insensitive 5
ANJ: ANJEA
ANX: ANXUR
AFB: AUXIN-SIGNALING F-BOX
BSU1: BRI1-suppressor 1
BIK1: Botrytis-induced kinase 1
BSK: BR signaling kinase
BL: Brassinolide
BRI1: BRASSINOSTEROID INSENSITIVE1
BAK1: BRASSINOSTEROID INSENSITIVE1-ASSOCIATED KINASE1
BAK1: BRI1-associated kinase1
BUPS: BUDDHA’S PAPER SEAL
CaM: Calmodulin
CrRLK1L: *Catharanthus roseus* receptor-like kinase 1-like
CWI: Cell wall integrity
CDG1: Constitutive differential growth 1
COR: Coronatine
CRPs: Cysteine-rich peptides
EFR: EF-Tu RECEPTOR
EIL1: EIN3-LIKE1
ER: Endoplasmic reticulum
EIN3: Ethylene insensitive 3
EIX: ETH-inducing xylanase
ECD: Extracellular domain
F-RALF: *F. oxysporum* secreted RALF-like
FER: FERONIA
FLS2: FLAGELLIN-SENSING 2
GSK3: Glycogen synthase kinase 3
GEFs: Guanine nucleotide exchange factors
GTPase: Guanosine triphosphatase
H ^+^ -ATPase 2, AHA2: H^+^-adenosine triphosphatase 2
HERK1: HERCULES RECEPTOR KINASE 1
KD: Kinase domain
LRR: Leucine-rich repeat
LRXs: Leucine-rich repeat extensins
LRE: LORELEI
LLGs: LORELEI-LIKE GPI-ANCHORED PROTEINS
MRI: MARIS
MAPK: Mitogen-activated protein kinases
NTA: NORTIA
PBL: PBS1-LIKE
PMEs: Pectin methylesterases
PHR1: PHOSPHATE STARVATION RESPONSE 1
PHOT1: PHOTOTROPIN 1
P1BS: PHR1-binding site sequence
PIN2: PIN-FORMED2
PCP-Bs: POLLEN COAT PROTEIN B-class peptides
PKs: Protein kinases
Pro-RALFs: RALF propeptides
ROS: Reactive oxygen species
RLKs: Receptor-like kinases
RLPs: Receptor-like proteins
ROP11: Rho of Plant 11
RIPK: RPM1-induced protein kinase
SAM: S-adenosylmethionine
SA: Salicylic acid
S1P: SITE-1 PROTEASE
TOE1: TARGET OF EAT1
TOR: TARGET OF RAPAMYCIN
TIR1: TRANSPORT INHIBITOR RESPONSE1
YUC: YUCCA
