# Supplementary material for: How a single receptor-like kinase exerts diverse roles: lessons from FERONIA
Source: Mol Hortic. 2022 Nov 18;2:25. doi: 10.1186/s43897-022-00046-9 (PMC10515002; doi:10.1186/s43897-022-00046-9)
Supplement: Supplementary file 1 — Additional file 1: Additional Table 1. Function of FER with corresponding ligands and coreceptors. [file 43897_2022_46_MOESM1_ESM.docx]

**Additional Table 1. Function of FER with corresponding ligands and coreceptors.**

| **function** | **ligand(activator)** | **ligand localization** | **coreceptor** | **description** | **mechanism** |
| --- | --- | --- | --- | --- | --- |
| Reproduction | RALF4 and RALF19 | pollen tubes | LRE | pollen tube rupture and sperm release | RALF4 and RALF19 triggered the formation of FER–LORELEI-NTA complex to initiate Ca^2+^ spiking and further induce pollen tube rupture and sperm release(Gao et al., 2022) |
|  | RALF6/7/16/36/37 | pollen tubes | ANJ/HERK1 and LRE | polytubey block | RALF6, 7, 16, 36, and 37 peptide ligands activate FER-ANJ-HERK1 complexe to establish the polytubey block(Galindo‐Trigo et al., 2020; Zhong et al., 2022) |
|  | PCP-Bs, RALF23/33 | PCP-Bs: pollens  RALF23/33: stigmas | ANJ | Pollen-pistil recognition | PCP-Bs compete with RALF23/33 for binding to the FER-ANJ complex, leading to a decline of stigmatic ROS that facilitates pollen hydration(Liu et al., 2021) |
|  | pectin | ovular | \ | polytubey block | The FER-mediated presence of de-esterified pectin underlies prevention of the entrance of supernumerary pollen tubes into ovules(Duan et al., 2020) |
| Abiotic stress | pectin | roots | LLG1 | salt stress | The FER preceieves pectin signal and maintains CWI during salt stress(Zou et al., 2018) |
|  | LRX3/4/5 and RALF22/23 | roots | \ | salt stress | The LRXs associate with RALF22/23 to activate FER and transduce cell-wall signals to regulate plant growth and salt stress tolerance(Zhao et al., 2018). |
|  | P1BS containing RALF1/4/22/23/33/34 | roots | \ | phosphate starvation | PHR induced RALF expression and further inhibit plant immunity through FER pathway to enhance bacterial growth that help to alleviate phosphate starvation (Tang et al., 2022) |
|  | RALF1 | young leaves | \ | nitrogen starvation | RALF1-FER-RIPK complex interacts with and activates TOR signaling, promoting growth of true leaves in plants(Song et al., 2022). |
|  | \ | roots | \ | mechanically stimulation | FER regulates Ca^2+^ signaling in mechanically stimulated Arabidopsis(Shih et al., 2014) |
|  | \ | roots | \ | High temperature stress | FER is necessary for plant high temperature resistance in a RALF1 independent pathway(Kim et al., 2021) |
|  | RALFs | \ | \ | metal ion stress | FER offers a molecular link between metal ion stress, growth and the cell wall integrity(Richter et al., 2017) |
| biotic stress | RALFs with S1P site | leaves | \ | FLS2/EFR mediated PTI | RALFs inhibit immunity through restraining the interaction between FER and FLS2/EFR(Stegmann et al., 2017) |
| Cell growth | \ | \ | \ | phototropic cell growth | PHOT1 interacts with and phosphorylates FER and further phosphorylates phytochrome kinase substrates (PKSs) to mediate phototropic cell growth(Li et al., 2022) |
|  | RALF1/22/33/36 | roots | \ | root elongation | RALF1 functions through FER to inhibit root elongation(Gjetting et al., 2020; Li et al., 2022) |
|  | LRX3/4/5 and RALF1 | root tip vacuoles | \ | cell elongation | LRX3/4/5 interact with FER to sense RALF1 and regulate cellular elongation(Dünser et al., 2019) |
|  | \ | \ | \ | cotyledon greening | FER phosphorylates and destabilizes ABI5 to negatively mediate cotyledon greening(Wang et al., 2022a) |
|  | pectin | cotyledon epidermal pavement cells | \ | PC morphogenesis | FER binds pectin to regulate PC morphogenesis through ROP6/GTPase signaling pathway(Tang et al., 2022) |
| Hormone response | RALF1/22 | roots | \ | IAA response | RALF1-FER promotes YUC expression and thus auxin biosynthesis to induce the canonical TIR1/AFB transcriptional pathway for sustained root growth inhibition(Li et al., 2022). Moreover, FER is vital for polar auxin transport(Li et al., 2020). |
|  | RALF1 | roots | \ | ABA response | RALF1-FER pathway activates ABI2 phosphatase and inhibit ABA response through also initiates the GEF1/4/10-ROP11 pathway(Chen et al., 2016) |
|  | RALF23 | both roots and shoots | \ | JA response | FER inhibits JA and COR signaling by phosphorylating and destabilizing MYC2, thereby positively regulating immunity(Guo et al., 2018). |
|  | RALF1/23 | roots and hypocotyls | \ | BR response | RALFs function antagonistically with BR through FER pathway(Bergonci et al., 2014a; Bergonci et al., 2014b; Srivastava et al., 2009). |
| Molecular mechanisms | \ | \ | \ | ER body formation | FER negatively regulates transcription factor NAI1 to mediate ER body formation(Wang et al., 2022a) |
|  | \ | \ | \ | indolic glucosinolate biosynthesis | FER negatively regulates transcription factor NAI1 to mediate indolic glucosinolate biosynthesis(Wang et al., 2022a) |
|  | \ | \ | \ | autophagy | FER functions through TOR to negatively regulate autophagy(Wang et al., 2022b) |

Bergonci T, Ribeiro B, Ceciliato PHO, Guerrero-Abad JC, Silva-Filho MC, & Moura DS. Arabidopsis thaliana RALF1 opposes brassinosteroid effects on root cell elongation and lateral root formation. *EXBOTJ.* 2014a; 65, 2219-2230.

Bergonci T, Silva-Filho MC, & Moura DS. Antagonistic relationship between AtRALF1 and brassinosteroid regulates cell expansion-related genes. *Plant Signal Behav.* 2014b; 9, e976146-e976146.

Chen J, Yu F, Liu Y, Du C, Li X, Zhu S, Wang X, Lan W, Rodriguez PL, Liu X, Li D, Chen L, & Luan S. FERONIA interacts with ABI2-type phosphatases to facilitate signaling cross-talk between abscisic acid and RALF peptide in Arabidopsis. *Proc Natl Acad Sci U S A.* 2016; 113, E5519-E5527.

Duan Q, Liu M-CJ, Kita D, Jordan SS, Yeh F-LJ, Yvon R, Carpenter H, Federico AN, Garcia-Valencia LE, Eyles SJ, Wang C-S, Wu H-M, & Cheung AY. FERONIA controls pectin- and nitric oxide-mediated male–female interaction. *Nature.* 2020; 579, 561-566.

Dünser K, Gupta S, Herger A, Feraru MI, Ringli C, & Kleine-Vehn J. Extracellular matrix sensing by FERONIA and Leucine-Rich Repeat Extensins controls vacuolar expansion during cellular elongation in Arabidopsis thaliana. *EMBO J.* 2019; 38, e100353.

Galindo‐Trigo S, Blanco‐Touriñán N, DeFalco TA, Wells ES, Gray JE, Zipfel C, & Smith LM. CrRLK1L receptor‐like kinases HERK1 and ANJEA are female determinants of pollen tube reception. *EMBO Rep.* 2020; 21, e48466.

Gao Q, Wang C, Xi Y, Shao Q, Li L, & Luan S. A receptor-channel trio conducts Ca^2+^ signalling for pollen tube reception. *Nature (London).* 2022; 607, 534-536.

Gjetting SK, Mahmood K, Shabala L, Kristensen A, Shabala S, Palmgren M, & Fuglsang AT. Evidence for multiple receptors mediating RALF-triggered Ca2+ signaling and proton pump inhibition. *The Plant Journal.* 2020; 104, 433-446.

Guo H, Nolan TM, Song G, Liu S, Xie Z, Chen J, Schnable PS, Walley JW, & Yin Y. FERONIA Receptor Kinase Contributes to Plant Immunity by Suppressing Jasmonic Acid Signaling in Arabidopsis thaliana. *Curr Biol.* 2018; 28, 3316-3324.

Kim D, Yang J, Gu F, Park S, Combs J, Adams A, Mayes HB, Jeon SJ, Bahk JD, & Nielsen E. A temperature-sensitive FERONIA mutant allele that alters root hair growth. *Plant Physiology.* 2021; 185, 405-423.

Li C, Chen J, Li X, Zhang X, Liu Y, Zhu S, Wang L, Zheng H, Luan S, Li J, & Yu F. FERONIA is involved in phototropin 1-mediated blue light phototropic growth in Arabidopsis. *Journal of Integrative Plant Biology.* 2022; 64, 1901-1915.

Li E, Wang G, Zhang YL, Kong Z, & Li S. FERONIA mediates root nutating growth. *Plant J.* 2020; 104, 1105-1116.

Li L, Chen H, Alotaibi SS, Pěnčík A, Adamowski M, Novák O, & Friml J. RALF1 peptide triggers biphasic root growth inhibition upstream of auxin biosynthesis. *Proceedings of the National Academy of Sciences.* 2022; 119, 1-e2121058119.

Liu C, Shen L, Xiao Y, Vyshedsky D, Peng C, Sun X, Liu Z, Cheng L, Zhang H, Han Z, Chai J, Wu H-M, Cheung AY, & Li C. Pollen PCP-B peptides unlock a stigma peptide-receptor kinase gating mechanism for pollination. *SCIENCE.* 2021; 372, 171-175.

Richter J, Ploderer M, Hauser MT, Mongelard G, & Gutierrez L. Role of CrRLK1L cell wall sensors HERCULES1 and 2, THESEUS1, and FERONIA in growth adaptation triggered by heavy metals and trace elements. *Frontiers in plant science.* 2017; 8, 1554-1554.

Shih H-W, Miller Nathan D, Dai C, Spalding Edgar P, & Monshausen Gabriele B. The Receptor-like Kinase FERONIA Is Required for Mechanical Signal Transduction in Arabidopsis Seedlings. *Current Biology.* 2014; 24, 1887-1892.

Song L, Xu G, Li T, Zhou H, Lin Q, Chen J, Wang L, Wu D, Li X, Wang L, Zhu S, & Yu F. The RALF1-FERONIA complex interacts with and activates TOR signaling in response to low nutrients. *Molecular Plant.* 2022; 15, 1120-1136.

Srivastava R, Liu JX, Guo H, Yin Y, & Howell SH. Regulation and processing of a plant peptide hormone, AtRALF23, in Arabidopsis. *Plant J.* 2009; 59, 930-939.

Stegmann M, Monaghan J, Smakowska-Luzan E, Rovenich H, Lehner A, Holton N, Belkhadir Y, & Zipfel C. The receptor kinase FER is a RALF-regulated scaffold controlling plant immune signaling. *SCIENCE.* 2017; 355, 287-289.

Tang J, Wu D, Li X, Wang L, Xu L, Zhang Y, Xu F, Liu H, Xie Q, Dai S, Coleman‐Derr D, Zhu S, & Yu F. Plant immunity suppression via PHR1‐RALF‐FERONIA shapes the root microbiome to alleviate phosphate starvation. *EMBO J.* 2022; 41, e109102.

Tang W, Lin W, Zhou X, Guo J, Dang X, Li B, Lin D, & Yang Z. Mechano-transduction via the pectin-FERONIA complex activates ROP6 GTPase signaling in Arabidopsis pavement cell morphogenesis. *Current Biology.* 2022; 32, 508-517.e503.

Wang P, Clark NM, Nolan TM, Song G, Bartz PM, Liao C-Y, Montes-Serey C, Katz E, Polko JK, Kieber JJ, Kliebenstein DJ, Bassham DC, Walley JW, Yin Y, & Guo H. Integrated omics reveal novel functions and underlying mechanisms of the receptor kinase FERONIA in Arabidopsis thaliana. *The Plant Cell.* 2022a; 34, 2594-2614.

Wang P, Clark NM, Nolan TM, Song G, Whitham OG, Liao C-Y, Montes-Serey C, Bassham DC, Walley JW, Yin Y, & Guo H. FERONIA functions through Target of Rapamycin (TOR) to negatively regulate autophagy. *Frontiers in plant science.* 2022b; 13. doi:10.3389/fpls.2022.961096.

Zhao C, Zayed O, Yu Z, Jiang W, Zhu P, Hsu CC, Zhang L, Andy Tao W, Lozano-Durán R, & Zhu JK. Leucine-rich repeat extensin proteins regulate plant salt tolerance in Arabidopsis. *Proc Natl Acad Sci U S A.* 2018; 115, 13123-13128.

Zhong S, Li L, Wang Z, Ge Z, Li Q, Bleckmann A, Wang J, Song Z, Shi Y, Liu T, Li L, Zhou H, Wang Y, Zhang L, Wu H-M, Lai L, Gu H, Dong J, Cheung AY, Dresselhaus T, & Qu L-J. RALF peptide signaling controls the polytubey block in Arabidopsis. *SCIENCE.* 2022; 375, 290-296.

Zou Y, Wang S, Zhou Y, Bai J, Huang G, Liu X, Zhang Y, Tang D, & Lu D. Transcriptional Regulation of the Immune Receptor FLS2 Controls the Ontogeny of Plant Innate Immunity. *Plant Cell.* 2018; 30, 2779-2794.
